# Precise Tuning of Polymeric Fiber Dimensions to Enhance the Mechanical Properties of Alginate Hydrogel Matrices

**DOI:** 10.3390/polym13132202

**Published:** 2021-07-02

**Authors:** Zehua Li, Amanda K. Pearce, Andrew P. Dove, Rachel K. O’Reilly

**Affiliations:** 1 School of Chemistry, University of Birmingham, Edgbaston, Birmingham B15 2TT, UK; ZXL798@student.bham.ac.uk (Z.L.); a.k.pearce@bham.ac.uk (A.K.P.); 2 Department of Chemistry, University of Warwick, Gibbet Hill Road, Coventry CV4 7AL, UK

**Keywords:** crystallization-driven self-assembly, calcium alginate hydrogel, cylindrical micelles

## Abstract

Hydrogels based on biopolymers, such as alginate, are commonly used as scaffolds in tissue engineering applications as they mimic the features of the native extracellular matrix (ECM). However, in their native state, they suffer from drawbacks including poor mechanical performance and a lack of biological functionalities. Herein, we have exploited a crystallization-driven self-assembly (CDSA) methodology to prepare well-defined one-dimensional micellar structures with controlled lengths to act as a mimic of fibrillar collagen in native ECM and improve the mechanical strength of alginate-based hydrogels. Poly(ε-caprolactone)-*b*-poly(methyl methacrylate)-*b*-poly(*N, N*-dimethyl acrylamide) triblock copolymers were self-assembled into 1D cylindrical micelles with precise lengths using CDSA epitaxial growth and subsequently combined with calcium alginate hydrogel networks to obtain nanocomposites. Rheological characterization determined that the inclusion of the cylindrical structures within the hydrogel network increased the strength of the hydrogel under shear. Furthermore, the strain at flow point of the alginate-based hydrogel was found to increase with nanoparticle content, reaching an improvement of 37% when loaded with 500 nm cylindrical micelles. Overall, this study has demonstrated that one-dimensional cylindrical nanoparticles with controlled lengths formed through CDSA are promising fibrillar collagen mimics to build ECM scaffold models, allowing exploration of the relationship between collagen fiber size and matrix mechanical properties.

## 1. Introduction

Fibrillar collagen is the basic building block for tissues and represents over 90% of the total abundant collagen family [1,2]. In vivo, most ECMs are formed by a hydrogel-like network of fibrous proteins comprised of fibrillar collagen, elastin, and a soft matrix combining proteoglycans and polysaccharides [3,4]. The physical properties and mechanical responses exhibited by ECMs are significantly influenced by their filamentous nature, which affects downstream cellular processes such as mechanotransduction [5,6]. During healthy aging, the fibrillar collagen content in many organs and tissues decreases, thus diminishing the integrity and strength of the ECM, which are fundamental properties for correct tissue and organ function [7,8]. For example, age-related skin wrinkling and stiffening of arteries are caused by a loss in integrity of fibrous proteins with age, which changes the mechanical properties of tissue [9]. In this regard, hydrogels are promising synthetic matrix materials to act as models to explore the effect of fibrillar collagen size on ECM mechanical properties. Indeed, not only can hydrogels reproduce ECM microenvironments, but also their mechanical properties [10]. Hence, by tailoring and designing the features of hydrogel-based systems, they can precisely satisfy specific biomedical applications [11,12].

Alginate is a widely used polysaccharide [13] and has been widely used as a matrix material in tissue engineering applications as a result of advantages such as low cytotoxicity, biocompatibility, non-immunogenicity, injectability, and low cost [14]. However, naturally-derived hydrogels suffer the drawback of being structurally weaker than synthetic hydrogels [15]. In addition, as alginate-derived hydrogels are hydrophilic and don’t possess a fibrillar structure or nanofillers, they have weak mechanical properties (i.e., strength and stiffness) [16,17] as well as a lack of biological functionalities [16]. In an effort to overcome this, a range of nanocomposite hydrogels have been explored through the combination of nanoparticles and natural biopolymers [18,19,20,21], where the mechanical properties of the natural hydrogel network were shown to be enhanced by the interaction between the added nanostructures and the biopolymer chains. Such interactions were capable of tuning the stiffness, porosity, and nanostructure of the hydrogel, thus improving its overall performance as a biomaterial [10,11,22,23,24]. Despite some promising achievements to date—for example, exploring the effect of nanoparticle morphology on the mechanical properties of hydrogels [11,24]—precise control of nanocomposite dimensions and this influence on hydrogel mechanical properties is rarely reported and hence remains an attractive and challenging area of research.

Generally, an ideal hydrogel biomaterial should satisfy the criteria of biocompatibility, biodegradability, and appropriate mechanical properties [25,26,27,28] such as maintaining a certain mechanical strength and stiffness even in a swollen state [29]. Furthermore, the features of incorporated nanoparticulate filament structures are particularly important for providing directionality and guidance within the composite material [30,31,32]. For instance, filamentous extracellular protein networks show stiffening with increasing shear strain, likely caused by collective rearrangements of the filaments [33,34]. However, contrary to filamentous networks, many synthetic fibrillar hydrogels are shear-thinning, whereby the viscosity decreases in response to increased shear strain as the hydrogel network breaks. This property is often exploited in tissue engineering, where the hydrogel is able to be injected into the body with a syringe [35], followed by rapid recovery of the mechanical properties [10]. This particular shear-thinning behavior means the process of using polymer fibers to improve the mechanical properties of nanocomposite hydrogels is complicated. In order to understand this complexity, modeling the role of filament-like nanoparticles and their dimensions in the enhancement of shear strength of alginate-based hydrogels can provide crucial insights into the relationship between fibrillar collagen size and the mechanical properties of the native ECM [36].

In order to effectively mimic the fibrous architecture of different lengths, precise control over the formation of synthetic polymer fibers is vital. The ability to tailor the dimensions of nanostructures [24] enables polymer fibers to exhibit intriguing similarities to natural fibers, and therefore access to fibrillar collagen mimics. Polymer self-assembly through solution crystallization of block copolymers, termed crystallization-driven self-assembly (CDSA), with a semi-crystalline core-forming block has the remarkable ability to precisely control the dimensions of polymer fibers and to date has been demonstrated for a range of polymer blocks including polyferrocenylsilane [37], poly(ε-caprolactone) [38], polylactide [39], polypeptoids [40], and oligo(p-phenylenevinylene) [41]. Previous reports have used biocompatible and biodegradable poly(ε-caprolactone) (PCL)-based copolymers to form cylindrical assemblies with controlled dimensions by epitaxial growth, which led to the manufacture of biocompatible fibrillar hydrogels in situ [38]. The influence of the polymer nanoparticle shape on material properties was further explored through morphology control, which was achievable through CDSA. When calcium-alginate hydrogels were blended with nanostructures of different morphologies, including 0D spheres, 1D cylinders, and 2D platelets, the mechanical strength of the nanocomposite hydrogels was enhanced to different degrees. The 2D nanostructures exhibited a significant increase compared to the 0D or 1D nanoparticle counterparts [24]. Although this work demonstrated that PCL-based cylindrical micelles could form hydrogels and that nanoparticle morphologies could direct the mechanical strength of nanocomposite hydrogels, to date, utilizing PCL-based cylindrical micelles of controllable lengths to precisely tune the mechanical properties of nanocomposite hydrogels has yet to be achieved. The outcome of such advances could help to further understand the relationship between nanofiller dimensions and nanocomposite hydrogel strength.

**Scheme 1:**
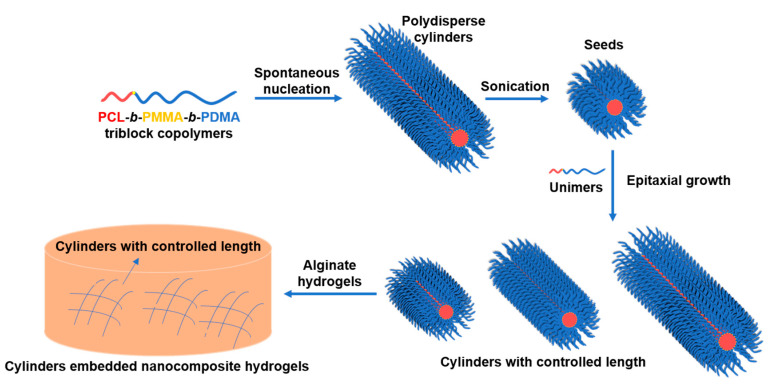
Formation of PCL-based cylindrical micelles with various lengths by epitaxial growth, and the fabrication of cylinder embedded nanocomposite hydrogels.

**Scheme 2:**
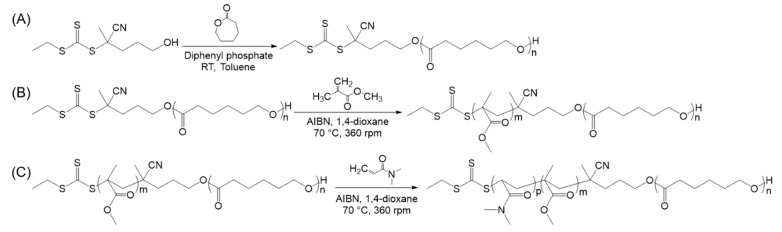
(**A**) Synthesis of PCL macro-CTA by ROP with a dual-headed CTA initiator and a DPP catalyst. (**B**) Synthesis of PCL-*b*-PMMA diblock copolymer by RAFT polymerization using AIBN as initiator. (**C**) Synthesis of PCL-*b*-PMMA-*b*-PDMA triblock copolymer by RAFT polymerization using AIBN as initiator.

Based on the above, in this study we aimed to introduce polymeric cylinders as a fibrillar structure within naturally-derived hydrogels and explore the influence of cylinder dimensions on material properties (Scheme 1). To achieve this, poly(ε-caprolactone)-*block*-poly(methyl methacrylate)-*block*-poly(*N, N*-dimethyl acrylamide) (PCL-*b*-PMMA-*b*-PDMA) triblock copolymers were synthesized (Scheme 2) and assembled into cylindrical micelles of controlled dimensions through CDSA. We demonstrated that the strength of nanocomposite hydrogels under shear could be improved when blended with 1D cylindrical nanoparticles, whereby the 500 nm cylindrical nanoparticles significantly increased the resistance of the nanocomposite hydrogels to flow under strain in comparison to their counterparts with other lengths. Overall, this work showed that nanoparticle cylinder length is one of the critical factors in precisely tuning the mechanical properties of alginate-based hydrogel networks, and that such nanocomposites show potential as collagen fiber mimics to explore the effect of dimensions on ECM mechanical properties.

## 2. Materials and Methods

### 2.1. Materials

Chemicals and solvents were purchased from Sigma-Aldrich, Acros, Fluka, TCI, Fisher Chemical, Alfa Aesar or VWR. ε-Caprolactone (ε-CL) monomer was distilled over calcium hydride before being stored in a glove box under an inert atmosphere. Diphenyl phosphate (DPP) was recrystallized once from dried CHCl_3_/Hexane (3:1) and dried over phosphorus pentoxide (P_2_O_5_) before use. (-)-Sparteine was dried over calcium hydride and distilled before use. 1,4-Dioxane, chloroform, methyl methacrylate (MMA), and *N,N*-dimethyl acrylamide (DMA) were purified by passing through basic alumina before use. 2,2′-Azobis(2-methylpropionitrile) (AIBN) was received from Molekula, recrystallized from methanol, and stored at 4 °C. Deuterated solvents were received from Apollo Scientific.

### 2.2. Typical Procedure for the ROP of PCL Homopolymers

In a nitrogen-filled glove box, solutions of DPP (49 mg, 0.20 mmol) in dry toluene (2 mL) and dual-headed CTA (52 mg, 0.21 mmol) in dry toluene (12.4 mL) were added to ε-CL (1.44 g, 12.62 mmol). After stirring for 11 h at room temperature, the solution was removed from the glove box, precipitated three times into ice-cold diethyl ether and collected by centrifugation. The resultant yellow polymer was dried under vacuum over phosphorus pentoxide for 2 days. The products were analyzed by SEC chromatograms and it was ensured there were no shoulders or tails in both sides of high or low molecular weight before proceeding with RAFT polymerizations and self-assembly. ^1^H NMR (300 MHz, CDCl_3_) δ/ppm: 4.06 (t, 100 H, CH_2_OCO), 3.65 (t, 2 H, CH_2_OH), 2.30 (t, 100 H, OCOCH_2_), 1.73–1.33 (m, 330 H, OCO(CH_2_)_5_OH), *M*_n_ = 6.2 kg mol^−1^, DP = 52. SEC chromatograms (DMF, PMMA standard): *M*_n_ = 13.1 kg mol^−1^, *M*_w_ = 14.3 kg mol^−1^, *Đ*_M_ = 1.09.

### 2.3. Typical Procedure for the Synthesis of PCL-b-PMMA Diblock Copolymers

PCL_50_ (500 mg, 0.08 mmol), MMA (420 mg, 4.20 mmol), and AIBN (1.38 mg, 8.39 × 10^−3^ mmol) were dissolved in 1,4-dioxane (1.40 mL) and placed in an ampoule. The solution was then freeze-pump-thawed three times and heated for 5 h at 70 °C. The reaction was quenched by immersion of the ampoule in the ice bath, and the polymer was precipitated in ice-cold methanol three times before being dried under vacuum and analyzed. ^1^H NMR (400 MHz, CDCl_3_) δ/ppm: 4.06 (t, 100 H, CH_2_OCO), 3.59 (45 H, COOCH_3_), 2.30 (t, 100 H, OCOCH_2_), 1.91–1.81 (2 m, 8 H, CCH_2_, PMMA), 1.72–1.33 (m, 305 H, OCO(CH_2_)_5_OH), 1.02–0.83 (m, 36 H, CH_3_, PMMA), *M*_n_ = 8.2 kg mol^−1^, DP = 20. SEC chromatograms (DMF, PMMA standard): *M*_n_ = 15.9 kg mol^−1^, *M*_w_ = 17.4 kg mol^−1^, *Đ*_M_ = 1.10.

### 2.4. Typical Procedure for the Synthesis of PCL-b-PMMA-b-PDMA Triblock Copolymers

PCL_50_-*b*-PMMA_20_ (150 mg, 0.02 mmol), DMA (396 mg, 4.00 mmol), and AIBN (0.3 mg, 1.83 × 10^−3^ mmol) were dissolved in 1,4-dioxane (316 μL) and placed in an ampoule. The solution was then freeze-pump-thawed three times and heated for 1 h at 70 °C. The reaction was quenched by immersion of the ampoule in the ice bath, and the polymer was precipitated in ice-cold diethyl ether three times before being dried under vacuum and analyzed. ^1^H NMR (400 MHz, CDCl_3_) δ/ppm: 4.04 (t, 100 H, CH_2_OH), 3.58 (45 H, COOCH_3_), 3.10–2.44 (m, 889 H, N(CH_3_)_2_, CHCH_2_, PDMA), 2.28 (t, 100 H, OCOCH_2_), 1.79–1.23 (m, 674 H, OCO(CH_2_)_5_OH (PCL), CCH_2_ (PMMA), CHCH_2_ (PDMA)), 1.02–0.83 (m, 48 H, CH_3_, PMMA), *M*_n_ = 27.6 kg mol^−1^, DP = 196. SEC chromatograms (DMF, PMMA standard): *M*_n_ = 34.2 kg mol^−1^, *M*_w_ = 38.5 kg mol^−1^, *Đ*_M_ = 1.12.

### 2.5. Typical Crystallization-Driven Self-Assembly Method for the Self-Nucleation of PCL Block Copolymers

As a typical procedure of self-assembly conditions, PCL-*b*-PMMA-*b*-PDMA triblock copolymer (25 mg) was added to 5 mL of ethanol (5.0 mg mL^−1^) in a vial. The samples were heated to 70 °C or 90 °C without stirring for 3 h before cooling to room temperature. Samples were imaged after 2 weeks of aging at room temperature.

### 2.6. Typical Gel Formation of Nanocomposite Calcium Alginates

Alginate gels were prepared at 1.5 wt. % sodium alginate. Before use, sodium alginate (19.9 mg, 0.1 mmol) was heated in the water to 70 °C for 1 h to aid dissolution and cooled to room temperature. Micelles were dispersed in the water for 2 h before stirring with calcium carbonate (5.0 mg, 0.05 mmol), followed by addition to the sodium alginate solution and vortexing for 1 min. After the addition of D-glucono-δ-lactone (GDL) (17.8 mg, 0.1 mmol), the gel was again vortexed for one minute before incubating at room temperature for two days.

## 3. Results and Discussion

### 3.1. Triblock Copolymer Synthesis and Characterization

To investigate the effect of polymer fiber dimensions on their ability to enhance the mechanical properties of hydrogels, cylindrical micelles with controlled lengths were prepared. According to previous literature, PCL block copolymers are able to produce cylindrical nanostructures by crystallization-driven self-assembly methodologies [42,43,44], where the cylinder length is controlled by epitaxial growth [24]. To overcome the disassembly of cylindrical micelles in water, which was attributed to the swelling of the corona block and subsequent fracture by the stress induced to the crystalline core, a glassy, highly hydrophobic polymer block was used to protect the PCL core. Therefore, following this strategy, poly(ε-caprolactone)-*block*-poly(methyl methacrylate)-*block*-poly(*N, N*-dimethyl acrylamide) (PCL-*b*-PMMA-*b*-PDMA) triblock copolymers were prepared.

The PCL-based triblock copolymers were synthesized using a combination of ring-opening polymerization (ROP) of ε-caprolactone (ε-CL) and reversible addition-fragmentation chain transfer (RAFT) polymerization of methyl methacrylate (MMA) and *N, N*-dimethyl acrylamide (DMA), respectively. First, the ROP of ε-CL was performed in a nitrogen-filled glove box at room temperature (RT) using a dual-headed initiator/chain transfer agent (CTA) and diphenyl phosphate (DPP) catalyst in dry toluene (Scheme 2A). The successful synthesis of PCL was indicated by proton nuclear magnetic resonance (^1^H NMR) spectroscopy, where the methylene resonances of the repeat units were observed at *δ* = 4.06 ppm and *δ* = 2.30 ppm, while the end group signal was at *δ* = 3.65 ppm (Figure S1). The degree of polymerization (DP) was calculated by end-group analysis comparing the integrations of characteristic signals, giving a DP of 55 and thus an *M*_n_ of 6.5 kg mol^−1^ (Table S1). Analysis of the PCL homopolymer by size exclusion chromatography (SEC) in tetrahydrofuran (THF) eluent revealed a narrow dispersity (*Đ*_M_ = 1.06, PMMA standards), which indicated a well-controlled polymerization, as well as no transesterification as detected by MALDI ToF mass spectrum (Figure S5). Importantly, the SEC chromatogram from the UV-Vis detector at *λ* = 309 nm confirmed the retention of the trithiocarbonate end group on the PCL homopolymer.

In order to prevent disassembly of the cylindrical micelles in water and subsequent rapid polymer precipitation, MMA was chosen as a hydrophobic RAFT compatible monomer to serve as an interfacial block before incorporation of the DMA hydrophilic block. The RAFT polymerization of MMA was carried out in 1,4-dioxane at 70 °C using the PCL macro-CTA as a RAFT agent (Scheme 2B). The reaction reached 40% monomer conversion after 5 h, and then purification was performed by precipitation into ice-cold methanol to obtain the PCL-*b*-PMMA diblock copolymer. The ^1^H NMR spectroscopic analysis revealed a DP of 19 and thus an *M*_n_ of 8.4 kg mol^−1^, using the methyl resonances of the PMMA repeat units at *δ* = 3.59 ppm and *δ* = 1.02 ppm (Figure S2). The subsequent RAFT polymerization of DMA was finally undertaken using the trithiocarbonate end group of the PCL-*b*-PMMA macro-CTA in 1,4-dioxane at 70 °C (Scheme 2C). The reaction reached 71% monomer conversion as determined by ^1^H NMR spectroscopy after 24 h, followed by quenching the polymerization and precipitation in ice-cold diethyl ether. The successful synthesis of the PCL-*b*-PMMA-*b*-DMA triblock copolymer was confirmed by ^1^H NMR spectroscopy (Figure S3), where the methyl resonances of the DMA repeat unit were observed at *δ* = 3.10–2.44 ppm, giving a calculated DP of 196 and an *M*_n_ of 27.6 kg mol^−1^. SEC analysis with DMF as eluent showed a narrow molecular weight distribution with a clear shift in molecular weight after each block and no observable evidence of low molecular weight species (*Đ*_M_ = 1.12, PMMA standards, Figures S4 and S5B,C).

### 3.2. Crystallization-Driven Self-Assembly for the Production of Cylindrical Micelles

**Figure 1:**
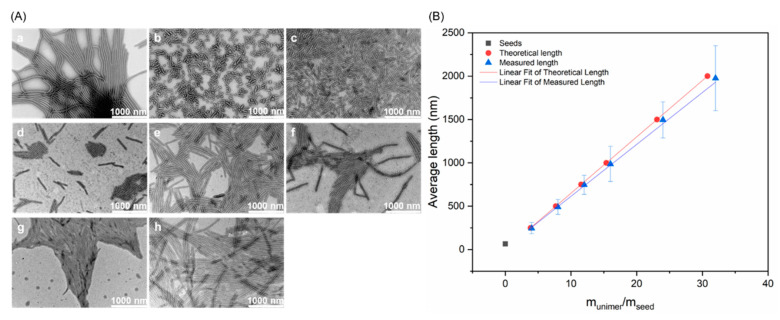
(**A**) TEM micrographs of (**a**) polydisperse cylindrical and (**b**) monodisperse seed micelles. Epitaxial growth of cylinders targeting (**c**) 250 nm, (**d**) 500 nm, (**e**) 750 nm, (**f**) 1000 nm, (**g**) 1500 nm, and (**h**) 2000 nm length values. 1% uranyl acetate was used as a negative stain. Scale bar = 1 μm. (**B**) Linear epitaxial growth of PCL_52_-*b*-PMMA_20_-*b*-PDMA_196_ cylinders with narrow length dispersity (blue triangles, error bars represent the standard deviation (σ) of the length distribution) in comparison to the theoretical length (red circles). The cylindrical micelles were grown from ca. 65 nm seeds.

To investigate the effect of nanoparticle dimensions on the mechanical strength of nanocomposite hydrogels, cylindrical micelles with different lengths were prepared and subsequently blended into alginate hydrogels to mimic the native ECM with a fibrillar structure. Initially, polydisperse 1D cylindrical micelles were prepared from the poly(ε-caprolactone)-based block copolymers using the spontaneous nucleation method as previously reported [38] (Scheme 1, Figure 1Aa). The crystalline nature of the PCL-based cylindrical micelles was confirmed using Wide Angle X-ray Scattering (WAXS) (Figure S6), where 2theta (2θ) peaks of crystalline PCL were observed at ca. 21° and 24° [45,46]. In order to transform the polydisperse cylinders into micelles of controlled dimensions, a living CDSA method was employed. In this process, crystalline seed micelles of uniform size are obtained through probe sonication of the polydisperse cylinders, which can then serve as initiation sites for further seeded growth of free polymer (unimer) in solution. With the controlled addition of polymer unimers, cylindrical micelles with low dispersity and precise length can be obtained, analogous to a living polymerization.

Spontaneously nucleated polydisperse micrometer-long cylinders (Figure 1Aa) were diluted in solution to 0.5 mg mL^−1^ and subjected to probe sonication to prepare seed micelles. In order to restrain undesired crystallization caused by local high temperatures, the sonication was performed under a controlled temperature of 0 °C, and the total time of 20 min was divided into 10 rounds of 2 min, with the micelle solution left cooling in the ice bath for at least 10 min between sonication rounds. Finally, uniform crystalline seeds were obtained with a number average length of 65 nm (Figure 1Ab,B).

For the epitaxial growth step, a unimer solution was prepared by dissolving the PCL_50_-*b*-PMMA_20_-*b*-PDMA_200_ triblock copolymers in tetrahydrofuran (THF) to form a 100 mg mL^−1^ solution. THF was chosen for this process as it is a good solvent for both blocks and also miscible with ethanol, which was the chosen corona-selective solvent. Different length cylindrical micelles were targeted by employing different unimer to seed ratios, whereby the unimer THF solution was added at the desired ratio into a 0.5 mg mL^−1^ seed micelle solution, followed by solvent evaporation at room temperature (RT) to obtain stable structures in ethanol. Controlled linear epitaxial growth was visually observed, with the longer cylinder solutions being more turbid, and further confirmed by TEM imaging. The analysis showed that monodisperse cylindrical micelles with precise micrometer lengths had been achieved, and that these were in agreement with predicted lengths (Figure 1Ac–h,B, Table 1). Indeed, the length displayed by the cylinders was proportional to the amount of unimer added (Table 1), which confirmed the controlled nature of the epitaxial growth process (Figure S7). The monodisperse cylindrical micelles were subsequently transferred into a pure aqueous phase by dialysis against water for 72 h. Importantly, the nanoparticles were confirmed to still exist as stable cylindrical nanostructures without disassembly or degradation after the dialysis process (Figure S8), therefore giving unprecedented access to collagen fiber mimics.

We next sought to investigate the relationship between the length of polymer fibers and mechanical strength of the nanocomposite hydrogels. Alginate was chosen as the hydrogel model matrix material in this work due to its high biocompatibility, low cost, facile processing technology, and ubiquity in translational medicine. Alginate is an anionic polysaccharide which forms a hydrogel structure through crosslinking with cationic substances, typically calcium [47,48]. In order to exclude any influences of surface charge on hydrogel formation, we first measured the ζ-potential of the PCL_50_-*b*-PMMA_20_-*b*-PDMA_200_ triblock copolymer cylinders. This was found to be ca. −4.34 mV, and thus could be considered approximately neutral [49] and as such avoid any additional effects of micelle surface charge on crosslinking.

Calcium-crosslinked alginate hydrogels (without cylinders) were used as a control group and were prepared by the combination of sodium alginate (1.5 wt %), calcium carbonate (CaCO_3_, 0.5 eq.), and D-glucono-δ-lactone (GDL, 1.0 eq.). The low solubility of CaCO_3_ relative to other crosslinking agents such as CaCl_2_ or CaSO_4_·2H_2_O allowed for a slower gelation process. In turn, this CaCO_3_-GDL system ensured uniform dispersion of calcium throughout the gel system before crosslinking occurred, preventing a heterogeneous structure which can lead to weak mechanical properties with low reproducibility [48]. Following establishment of the protocol for control calcium-crosslinked alginate hydrogels, the precise length polymer fibers were then incorporated during the crosslinking process, and TEM imaging was used to confirm that the cylinders remained intact during the mixing and vortexing process (Figure S9). The calcium concentration was kept constant, and different equivalents of monodisperse PCL_50_-*b*-PMMA_20_-*b*-PDMA_200_ cylindrical micelles were added at RT.

The mechanical strength of the resultant nanocomposite hydrogels was characterized through oscillatory rheology measurements. A control was performed in each dataset to ensure that the trends were not affected by slight variations in sample preparation such as lab temperature, which could cause a small shift in the magnitude of the theoretical moduli between data sets. For all samples, a gel-like behavior was confirmed by comparing the storage and loss modulus (*G’* and *G”*), whereby *G’* was higher than *G*” for the entire range of frequency sweep (Figure S10). Amplitude sweep tests also provided strain-dependent information. Specifically, the broad linear-viscoelastic (LVE) region and the hydrogel network breakdown were observed as the strain increased. In the LVE region, no substantial change in *G*’ was observed when increasing the nanoparticle content, which indicated the low embrittlement of the nanocomposite hydrogels as a consequence of the addition of fillers. At high strain, the strain at the flow point (*τ*_f_) was obtained (i.e., intersection of the curves for G’ and *G*”), with the value of shear stress being determined at the crossover point (*G*’ = *G*”, Figure S11).

**Figure 2:**
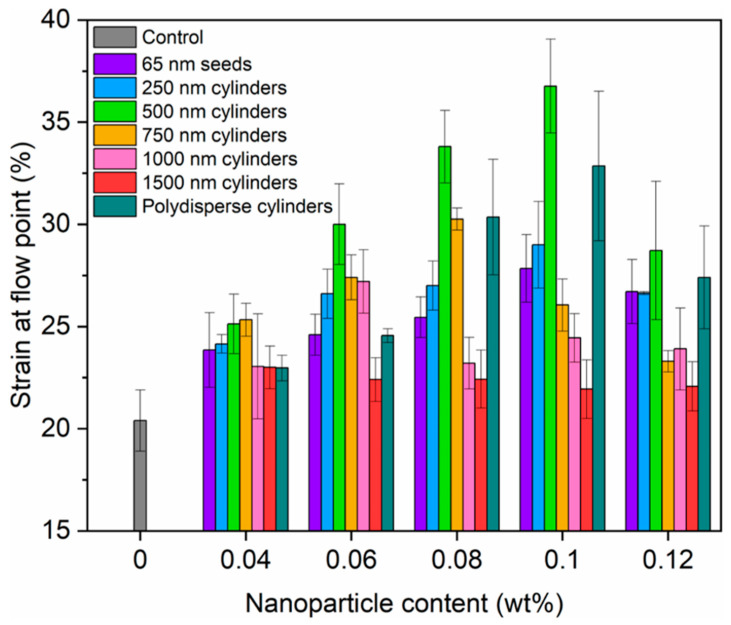
Histogram comparing the strain values at the flow point for nanocomposite hydrogels incorporating cylindrical micelles at different wt %. Error bars represent the standard deviation of the data.

The strain-dependent response of the control alginate hydrogel without nanoparticles showed a low strain value (*ca.* 20%) at the flow point. After the addition of 0.04, 0.06, 0.08, 0.10, and 0.12 wt % cylindrical micelles, the flow strain increased in comparison to the control group (Figure 2, Table S2). Hence, polymer cylindrical micelles with different lengths could enhance the mechanical strength of the nanocomposite calcium-crosslinked alginate hydrogels. In particular, the strain at the flow point of the nanocomposite hydrogel embedded with 500 nm PCL_50_-*b*-PMMA_20_-*b*-PDMA_200_ cylindrical micelles distinctly increased up to ca. 37% (at 0.10 wt %). We hypothesized that this phenomenon was related to the pore size of the calcium-crosslinked alginate hydrogels, with the 500 nm cylinders better able to pack into the pores of the hydrogels. The strain values at the flow point were found to increase and then decrease with the cylinder wt % for each cylinder length—although the maximum value was obtained at different wt % for different lengths of cylinder. Notably, this behavior was not observed with cylinders of 1000 nm and 1500 nm lengths, for which no significant enhancement was observed. Furthermore, when the cylindrical micelles’ nanoparticle content reached 0.12 wt %, the hydrogel strain value decreased for all systems, which was ascribed to steric hindrance [24,50]. In this case, we postulate that surplus cylinders were unable to pack inside the pores of the hydrogel network, thus reducing homogeneity and, consequently, disrupting hydrogel formation and yielding poorer mechanical features. Finally, polydisperse cylinders were prepared by mixing equal masses of 250 nm, 500 nm, 750 nm, 1000 nm, and 1500 nm cylinders to investigate the extent to which mixed micelles could affect the mechanical properties of hydrogels. The polydisperse cylinders showed a robust enhancement of the hydrogel strain properties, which was second only to the 500 nm samples.

These results showed that the dimensions of added fibrillar nanoparticles play an essential role in tuning the mechanical strength of nanocomposite hydrogels. The addition of cylindrical nanoparticles less than 750 nm in length was able to enhance the strain at flow point for the alginate hydrogels; however, further increasing cylinder length to 1000 nm and 1500 nm resulted in no improvement in mechanical properties. We hypothesize that the short cylinders of 65 nm, 250 nm, 500 nm, and 750 nm lengths could provide an efficient contribution to the mechanical resistance of hydrogels under shear due to being readily distributed throughout the hydrogel network. However, when the cylinders were overly short (65 nm or 250 nm in length), the cylinder ends were not able to effectively connect the hydrogel network in the pores, thus limiting such improvement. In contrast, 1000 nm and 1500 nm cylinders hindered the enhancement, likely because of the steric hindrance of long cylinders. Importantly, our results showed that when cylinders of 500 nm in length were incorporated, the resistance of the hydrogels to shear could be substantially enhanced through uniform embedding of the polymer fibers, and therefore that cylindrical micelles that can fit best in the pores of hydrogels should be preferentially considered as additives.

## 4. Conclusions

In this work, the relationship between the length of polymer fibers and the strain at the flow point of nanocomposite calcium-crosslinked alginate hydrogels was studied. Monodisperse PCL_50_-*b*-PMMA_20_-*b*-PDMA_200_ cylindrical micelles were achieved through CDSA to give neutrally charged cylinders of 65 nm, 250 nm, 500 nm, 750 nm, 1000 nm, and 1500 nm in length, which were subsequently embedded within calcium-alginate hydrogel matrices. Oscillatory rheology measurements showed that cylinders of 750 nm in length and below, as well as a sample of polydisperse cylindrical micelles, were able to enhance the strain at flow point of the alginate hydrogels. When cylinders of 500 nm length were incorporated, the nanocomposite hydrogels showed an enhancement of strain at flow point by up to ca. 37%. In comparison, no significant improvement was observed for 1000 nm and 1500 nm samples. Overall, this work demonstrates that hydrogel strength can be enhanced through uniform embedding of polymer fibers in a size-dependent manner, and therefore that nanocomposite hydrogels show potential to mimic the native ECM as scaffolds for a wide range of biomedical applications in vivo, such as manufacturing artificial skin or tissues.

## Figures and Tables

**Table 1:** Length dispersity of cylinders formed by epitaxial growth of PCL_50_-*b*-PMMA_20_-*b*-PDMA_200_ cylindrical micelles.

Target (nm)	*L*_w_^a^ (nm)	*L*_n_^b^ (nm)	*L*_w_/*L*_n_
Seeds	68	65	1.05
250 nm	262	246	1.07
500 nm	506	491	1.03
750 nm	762	746	1.02
1000 nm	1029	988	1.04
1500 nm	1561	1532	1.02
2000 nm	1984	1911	1.04

Imaged by TEM; one hundred cylinders per sample were counted to measure the length of cylinders by ImageJ. ^a^ *L*_w_ = weight average length. ^b^ *L*_n_ = number average length.

## Data Availability

The data presented in this study are available from the corresponding author upon reasonable request.

## Supplementary Materials

The following are available online at https://www.mdpi.com/article/10.3390/polym13132202/s1. Figure S1. ^1^H NMR spectra (300 MHz, CDCl_3_) of PCL_52_ homopolymer. Figure S2. ^1^H NMR spectra (300 MHz, CDCl_3_) of PCL_52_-*b*-PMMA_20_ diblock copolymer. Figure S3. ^1^H NMR spectra (300 MHz, CDCl_3_) of PCL_52_-*b*-PMMA_20_-*b*-PDMA_196_ triblock copolymer. Figure S4. Overlaid (A) RI and (B) UV (λ = 309 nm) SEC chromatograms of PCL macro-CTA, PCL-*b*-PMMA diblock copolymer, and PCL-*b*-PMMA-*b*-PDMA triblock copolymer using DMF with 5 mM NH_4_BF_4_ as the eluent and PMMA standards. Figure S5. (A) MALDI ToF mass spectra of PCL_52_ homopolymers, which showed a *m/z* difference of 114.14 equivalent to a PCL repeat unit and, therefore, minimal transesterification. (B) DOSY NMR spectra (500 MHz, CDCl_3_) of PCL_52_-*b*-PMMA_20_ diblock copolymers. (C) DOSY NMR spectra (500 MHz, CDCl_3_) of PCL_52_-*b*-PMMA_20_-*b*-PDMA_196_ triblock copolymers. Figure S6. WAXS spectra obtained of nanoparticles prepared from PCL_52_-based triblock copolymers, exhibiting the 2θ peaks at *ca.* 21° and 24°. Figure S7. Diameter distribution of (a) polydisperse cylinders, (b) seeds, (c) 250 nm cylinders, (d) 500 nm cylinders, (e) 750 nm cylinders, (f) 1000 nm cylinders, (g) 1500 nm cylinders, and (h) 2000 nm cylinders. Figure S8. TEM micrographs of (a) polydisperse cylinders, (b) seeds, (c) 250 nm cylinders, (d) 500 nm cylinders, (e) 750 nm cylinders, (f) 1000 nm cylinders, (g) 1500 nm cylinders, and (h) 2000 nm cylinders in water. 1% uranyl acetate was used as a negative stain. Scale bar = 1 μm. Figure S9. Dynamic oscillatory frequency sweeps at 0.5% strain of calcium-crosslinked alginate hydrogels at 0.50 eq. calcium with 0 wt% (control), 0.04 wt%, 0.06 wt%, 0.08 wt%, 0.10 wt%, and 0.12 wt% PCL_50_-*b*-PMMA_20_-*b*-PDMA_200_ cylindrical micelles with different lengths, which included (a) 65 nm, (b) 250 nm, (c) 500 nm, (d) 750 nm, (e) 1000 nm, (f) 1500 nm, and (g) polydisperse cylinders. Figure S10. Strain-dependent oscillatory rheology measurements at 10 rad s^-1^ angular frequency (ω) of calcium-crosslinked alginate hydrogels at 0.50 eq. calcium with 0 wt% (control), 0.04 wt%, 0.06 wt%, 0.08 wt%, 0.10 wt%, and 0.12 wt% PCL_50_-*b*-PMMA_20_-*b*-PDMA_200_ cylindrical micelles with different lengths, which included (a) 65 nm, (b) 250 nm, (c) 500 nm, (d) 750 nm, (e) 1000 nm, (f) 1500 nm, and (g) polydisperse cylinders. Table S1. Polymer characterization data for PCL_52_-*b*-PMMA_20_-*b*-PDMA_196_ block copolymers.Table S2 Strain values at the flow point for nanocomposite alginate hydrogels enriched with different concentrations of PCL_50_-*b*-PMMA_20_-*b*-PDMA_200_ cylinders with different lengths. Data are presented as average ± standard deviation.
