# Supplementary material for: Precise Tuning of Polymeric Fiber Dimensions to Enhance the Mechanical Properties of Alginate Hydrogel Matrices
Source: Polymers (Basel). 2021 Jul 2;13(13):2202. doi: 10.3390/polym13132202 (PMC8271374; doi:10.3390/polym13132202)
Supplement: Supplementary file 1 [file polymers-13-02202-s001.zip › polymers-1256871-supplementary.pdf]

Supporting Information for

Precise tuning of polymeric fiber dimensions to enhance the mechanical properties of alginate hydrogel matrices

Zehua Li[1,2], Amanda K. Pearce[1], Andrew P. Dove[1*], and Rachel K. O'Reilly[1*]

Materials and additional methods

Materials. Chemicals and solvents were purchased from Sigma-Aldrich, Acros, Fluka, TCI, Fisher Chemical, Alfa Aesar or VWR. ε-Caprolactone (ε-CL) monomer was distilled over calcium hydride before being stored in a glove box under an inert atmosphere. Diphenyl phosphate (DPP) was recrystallized once from dried CHCl3/Hexane (3:1) and dried over phosphorus pentoxide (P_2O_5) before use. (-)-Sparteine was dried over calcium hydride and distilled before use. 1,4-Dioxane, chloroform, methyl methacrylate (MMA), and *N,N*-dimethyl acrylamide (DMA) were purified by passing through basic alumina before use. 2,2′-Azobis(2-methylpropionitrile) (AIBN) was received from Molekula, recrystallized from methanol, and stored at 4 °C. Deuterated solvents were received from Apollo Scientific.

Characterization methods

^{1}H nuclear magnetic resonance (NMR) spectroscopy. ^{1}H NMR spectra were recorded at 300 MHz or 400 MHz on a Bruker AV-300 or a Bruker AV-400 spectrometer respectively. All spectra were recorded in $CDCL_3$ unless otherwise stated. The chemical shifts are reported as δ in parts per million (ppm) and quoted downfield from the internal standard tetramethylsilane (δ = 0 ppm).

Diffusion-ordered spectroscopy (DOSY) NMR. DOSY NMR was performed on a Bruker AV-500 AVANCE spectrometer equipped with a 5 mm broadband observe (BBO) z-axis gradient probe, which generated nominal maximum field strengths of 53.5 G cm^{-1}. The measurement was carried out using stimulated echo and LED pulse sequences incorporating bipolar-gradient pulses for diffusion with a diffusion time of 100 ms and a LED delay of 5 ms. For each experiment, pulsed-field gradients with a duration of 2.5 ms and a recovery delay of 200 μs were applied respectively with increases from 5% to 95% of the maximum strength in 32 equally spaced steps. Experiments were performed on samples at a polymer concentration of 10 mg mL^{-1} in deuterated chloroform with active temperature regulation at 298 K. The DOSY spectrum was processed by the Bruker Topspin S3 software package (version 2.1).

Size exclusion chromatography (SEC). SEC in DMF was performed on an Agilent 1260 Infinity II LC system equipped with a Wyatt DAWN HELEOS II multi-angle laser light scattering (MALLS) detector, a Wyatt Optilab T-rEX differential refractive index detector, an Agilent 1260 Infinity II WR diode array detector, an Agilent guard column (PLGel 5 μM, 50 × 7.5 mm) and two Agilent Mixed-C columns (PLGel 5 μM, 300 × 7.5 mm). The mobile phase was DMF (HPLC grade) containing 5 mM NH_4BF_4 at 50 °C at flow rate of 1.0 mL min^{-1}. Number average molecular weights (Mn), weight average molecular weights (Mw) and dispersities ($Đ_M$ = M_w/M_n) were determined using Wyatt ASTRA v7.1.3 software against

poly(methyl methacrylate) (PMMA) standards. For molecular weight determination via MALLS, dn/dc values were either measured by differential refractometry or calculated from the SEC chromatograms assuming 100% mass elution from the columns.

Matrix-assisted laser desorption/ionization-time of flight (MALDI ToF) mass spectrometer. Mass spectra were obtained using a Bruker Ultraflex II MALDI ToF mass spectrometer. The MALDI ToF samples were prepared using trans-2-[3-(4-t-butyl-phenyl)-2-methyl-2-propenylidene] malononitrile (DCTB) as matrix and sodium trifluoroacetate (NaTFA) was used as cationization agent. The typical procedure is as follows: DCTB (2 μL, 10 mg mL^{-1} in tetrahydrofuran (THF)), sample (2 μL, 1 mg mL^{-1} in THF) and NaTFA (2 μL, 0.1 mg mL^{-1} in THF) were added to the MALDI ToF plate followed by solvent evaporation. The samples were measured in reflection ion mode and calibrated by SpheriCal (1200 - 8000 g mol^{-1}) standards.

Sonication Probe. Bandelin Sonopuls HD2200 sonication probe was performed in an ice bath, where sonication was carried out in cycles of 2 minutes sonication, followed by 10 minutes of cooling in an ice bath (0 °C). Samples were regularly taken at time points and analyzed by TEM. Seed micelles were obtained by 20 minutes sonication.

Transmission Electron Microscopy (TEM). TEM was performed using JEOL 2000FX or JEOL 2100FX at 200 kV. TEM solution was typically made up at 0.1 mg mL^{-1} in a solvent. Then, 10 μL of sample solution was dropped onto a carbon/formvar-coated copper grid placed on filter paper. After 10 minutes drying, 10 μL of a 1% uranyl acetate solution was dropped onto the grid and left to dry overnight. TEM images were analyzed by Image J. The number-average length (L_n), and weight-average length (L_w) were obtained by counting at least 100 particles for each sample. L_n and L_w were calculated by the following equations:

$$L_n = \frac{\sum_{i=1}^{n} N_i L_i}{\sum_{i=1}^{n} N_i}$$

$$L_w = \frac{\sum_{i=1}^{n} N_i L_i^2}{\sum_{i=1}^{n} N_i L_i}$$

where Li is the length each counted cylinders and N_i is the number of the cylinders with the length L_i.

Wide angle x-ray scattering (WAXS). WAXS was performed on a Panalytical X'Pert Pro MPD equipped with a Cu $K_{\alpha 1}$ hybrid monochromator as the incident beam optics. The typical procedure is placing 20 mg of dried particles on a 10 mm silica sample holder. Then, a standard "powder" 2θ-θ diffraction scan was carried out in the angular range from 5° to 40° 2θ at room temperature. The WAXS were processed by MDI Jade software to calculate crystallinity.

Zeta Potential. Zeta potentials were measured using a Malvern Zetasizer Nano ZS. Particles were prepared as a suspension at pH 6.0, and the zeta potentials were obtained by measuring the electrophoretic movement of the particles under an applied electric field at 25 °C. All values were taken as averages from 5 repeat measurements.

Rheological measurements. Oscillatory shear rheology measurements were performed on an Anton Paar MCR 302 equipped with a PP50 geometry. Temperature was controlled with a P-PTD 200/AIR Peltier and a P-PTD 200 hood. Sample was loaded directly and then was pressed

at 20 °C into a disc 0.5 mm thick before testing. Frequency sweeps were performed at 0.5% strain from 0.1 to 100 rad s^{-1} angular frequency (ω). Amplitude sweeps were performed at 10 rad s^{-1} ω from 0.01 to 100% strain. Results were recorded as an average of 3 consecutive runs at a constant temperature of 20 °C.

SUPPLEMENTARY DATA

Polymer Characterization Data

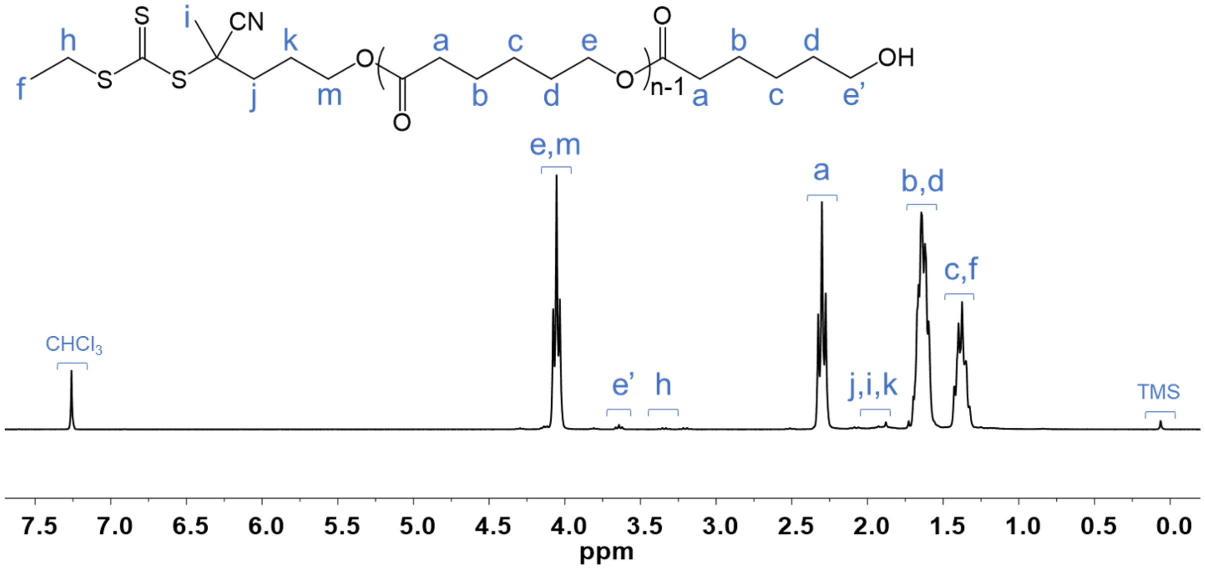

Figure S1. ^{1}H NMR spectra (300 MHz, $CDCl_3$) of PCL_{52} homopolymer.

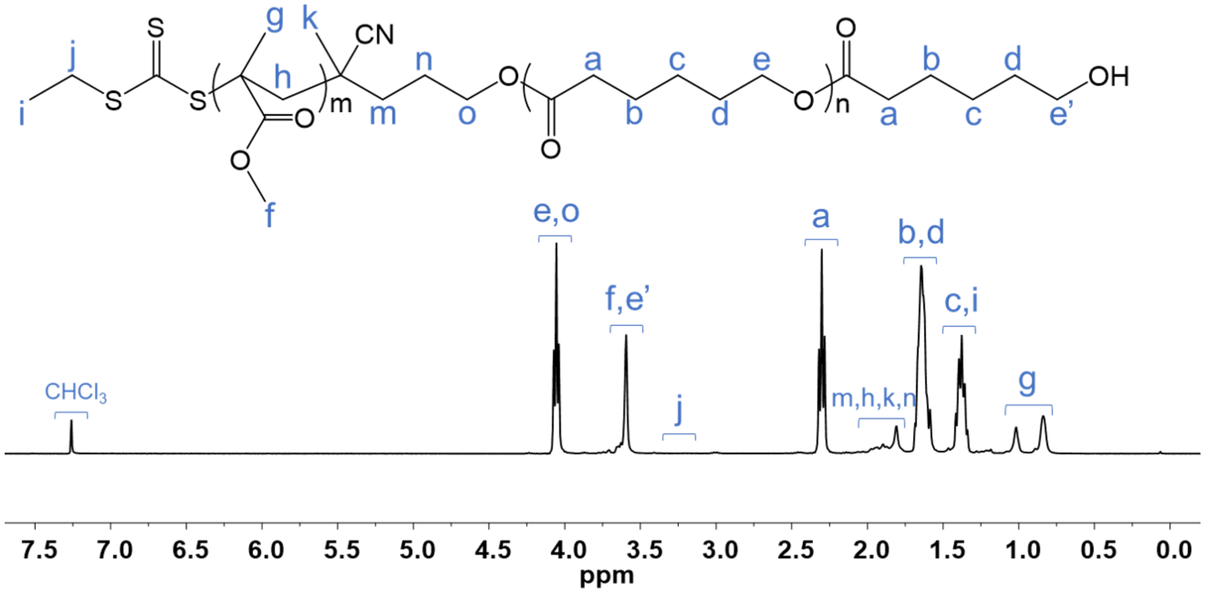

Figure S2. ^{1}H NMR spectra (300 MHz, $CDCl_3$) of PCL_{52}-*b*-$PMMA_{20}$ diblock copolymer.

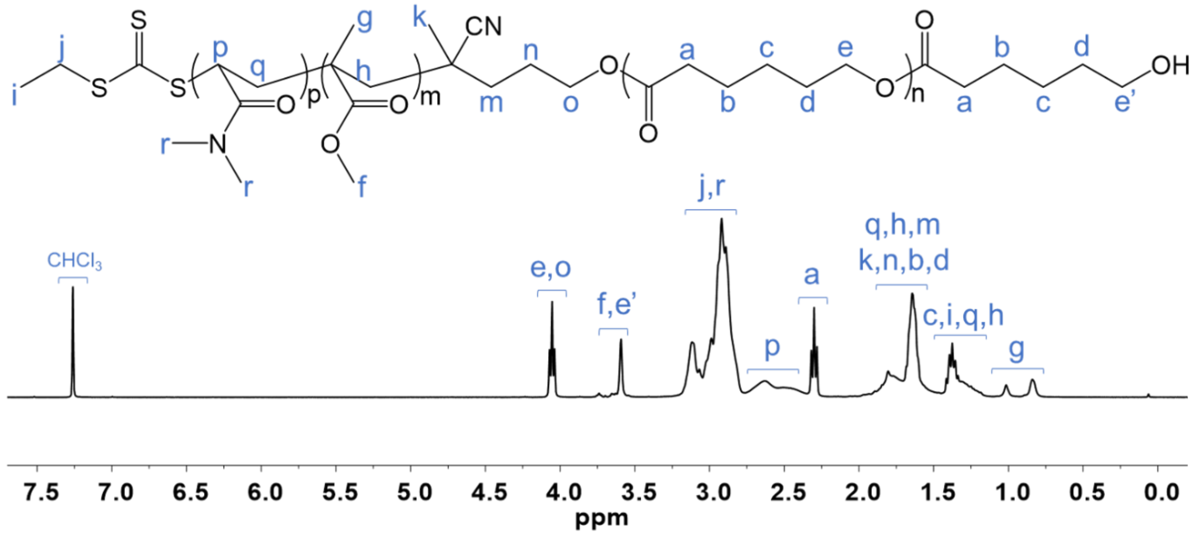

Figure S3. ^{1}H NMR spectra (300 MHz, $CDCl_3$) of PCL_{52}-*b*-$PMMA_{20}$-*b*-$PDMA_{196}$ triblock copolymer.

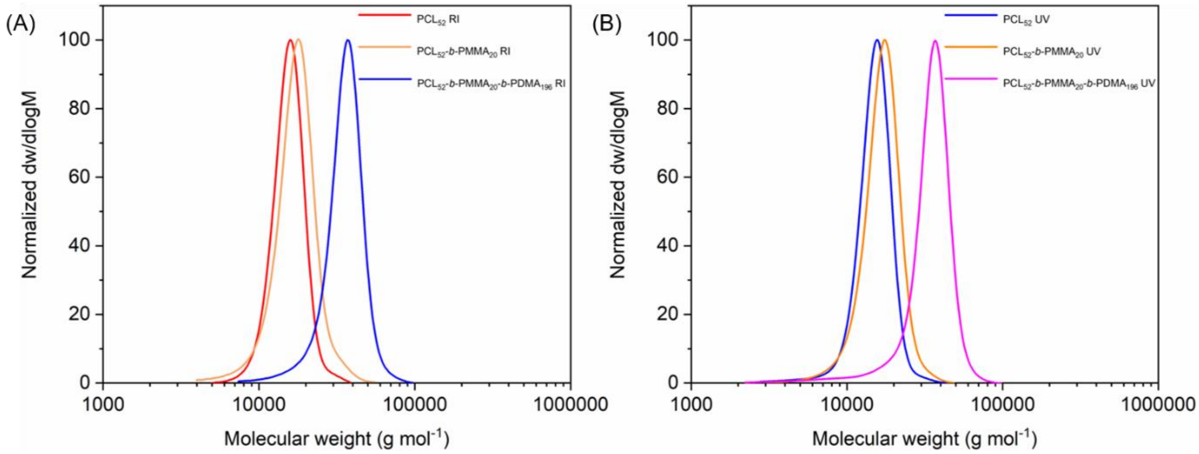

Figure S4. Overlaid (A) RI and (B) UV (λ = 309 nm) SEC chromatograms of PCL macro-CTA, PCL-*b*-PMMA diblock copolymer, and PCL-*b*-PMMA-*b*-PDMA triblock copolymer using DMF with 5 mM NH_4BF_4 as the eluent and PMMA standards.

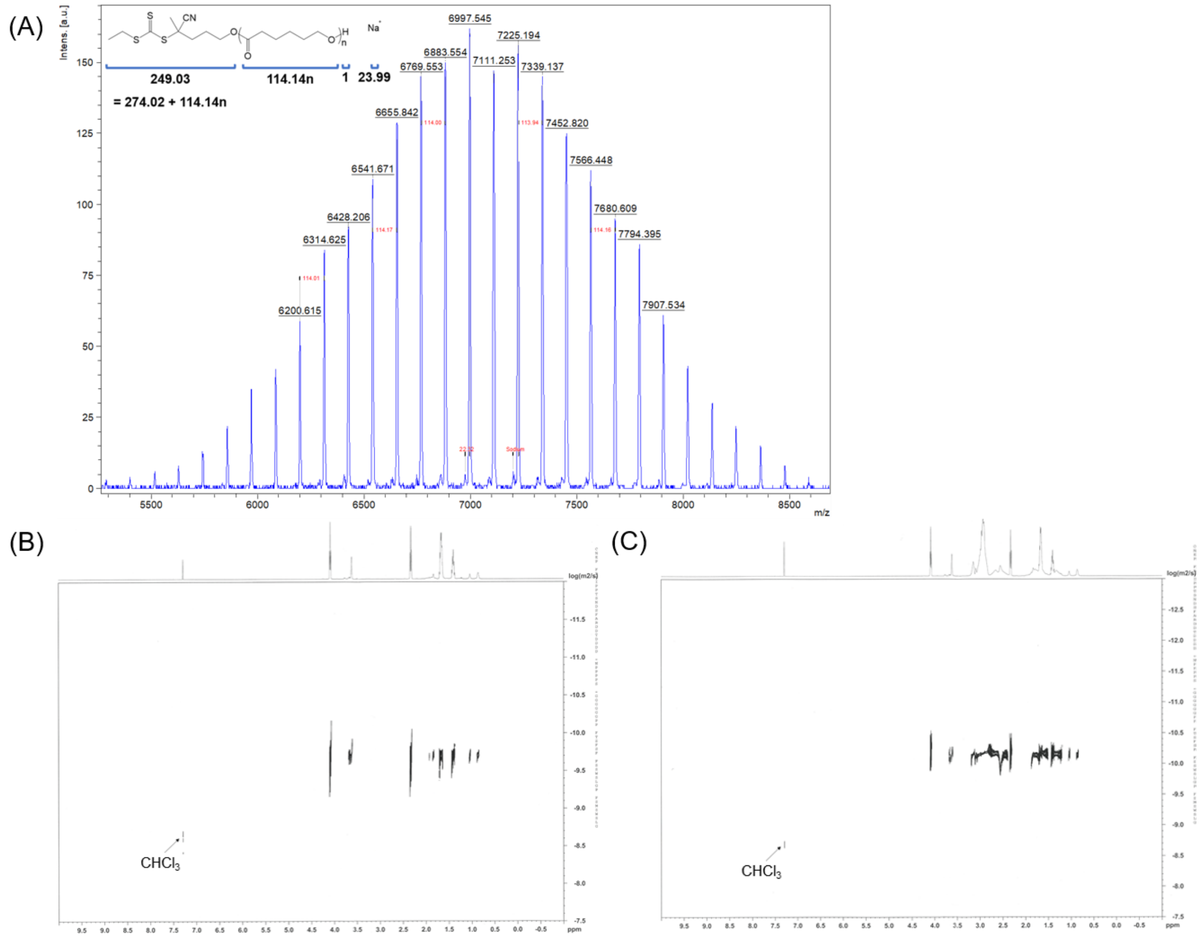

Figure S5. (A) MALDI ToF mass spectra of PCL_{52} homopolymers, which showed a m/z difference of 114.14 equivalent to a PCL repeat unit and, therefore, minimal transesterification. (B) DOSY NMR spectra (500 MHz, $CDCl_3$) of PCL_{52}-*b*-$PMMA_{20}$ diblock copolymers. (C) DOSY NMR spectra (500 MHz, $CDCl_3$) of PCL_{52}-*b*-$PMMA_{20}$-*b*-$PDMA_{196}$ triblock copolymers.

Table S1. Polymer characterization data for PCL_{52}-*b*-$PMMA_{20}$-*b*-$PDMA_{196}$ block copolymers.

Samples	DP_{PCL} [a]	DP_{PMMA} [a]	DP_{PDMA} [a]	M_n [a] (kg mol^{-1})	M_n [b] (kg mol^{-1})	M_w [b] (kg mol^{-1})	$Đ_M$ [b]
PCL	52	/	/	6.2	13.1	14.3	1.09
PCL-*b*-PMMA	52	20	/	8.2	15.9	17.4	1.10
PCL-*b*-PMMA-*b*-PDMA	52	20	196	27.6	34.2	38.5	1.12

[a] Measured by ^{1}H NMR spectroscopy in $CDCl_3$. [b] Measured by GPC analysis (DMF, RI, and UV309 detection).

Particle Characterization Data

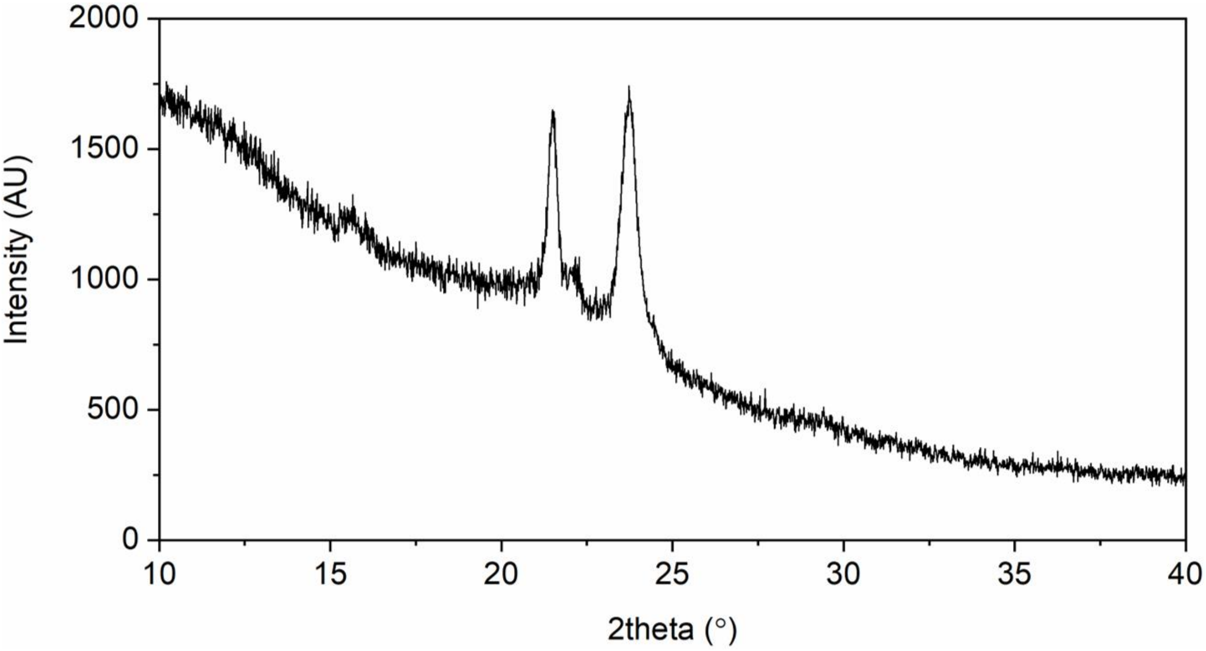

Figure S6. WAXS spectra obtained of nanoparticles prepared from PCL_{52}-based triblock copolymers, exhibiting the 2θ peaks at *ca.* 21˚ and 24˚.

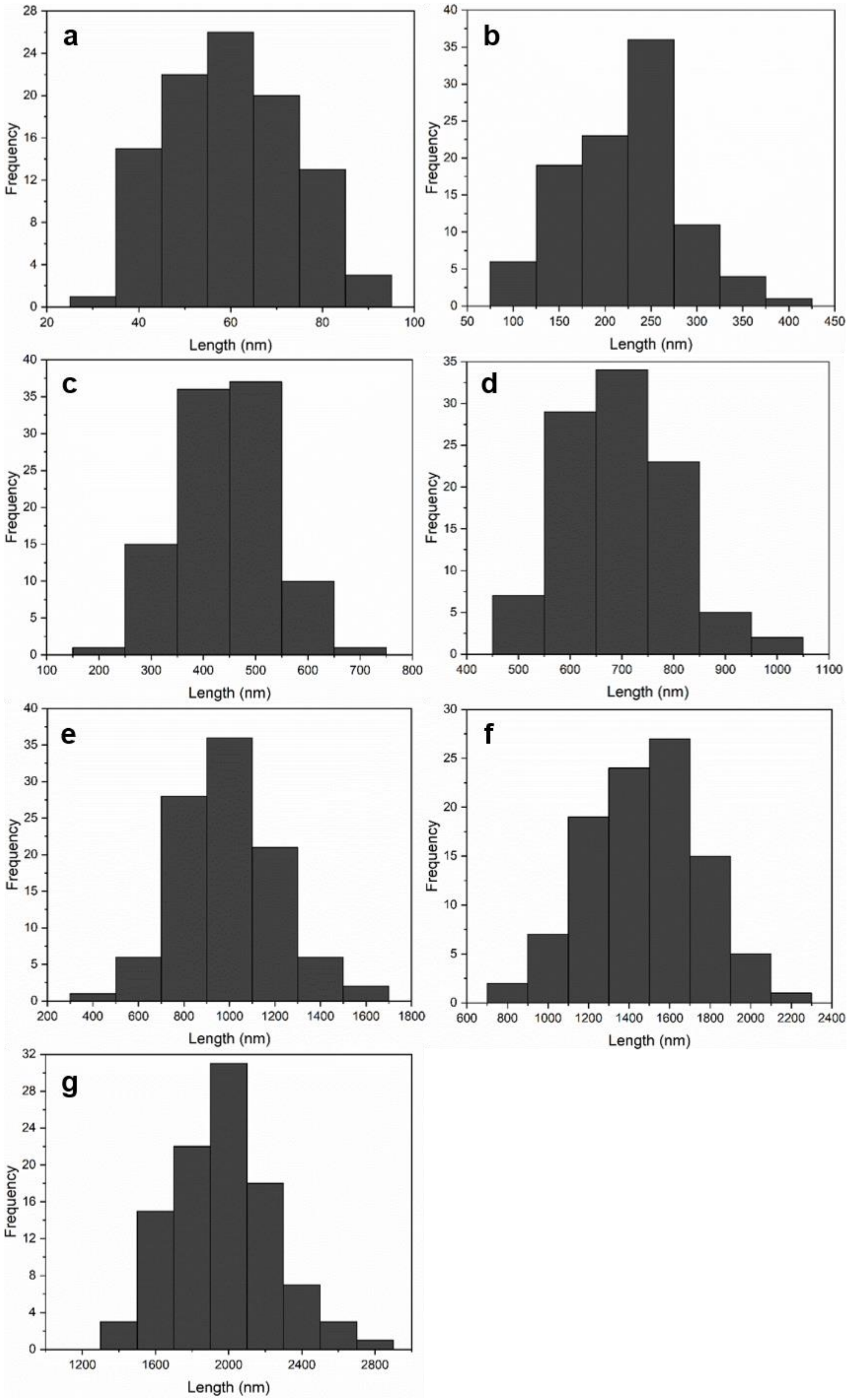

Figure S7. Diameter distribution of (**a**) polydisperse cylinders, (**b**) seeds, (**c**) 250 nm cylinders, (**d**) 500 nm cylinders, (**e**) 750 nm cylinders, (**f**) 1000 nm cylinders, (**g**) 1500 nm cylinders, and (**h**) 2000 nm cylinders.

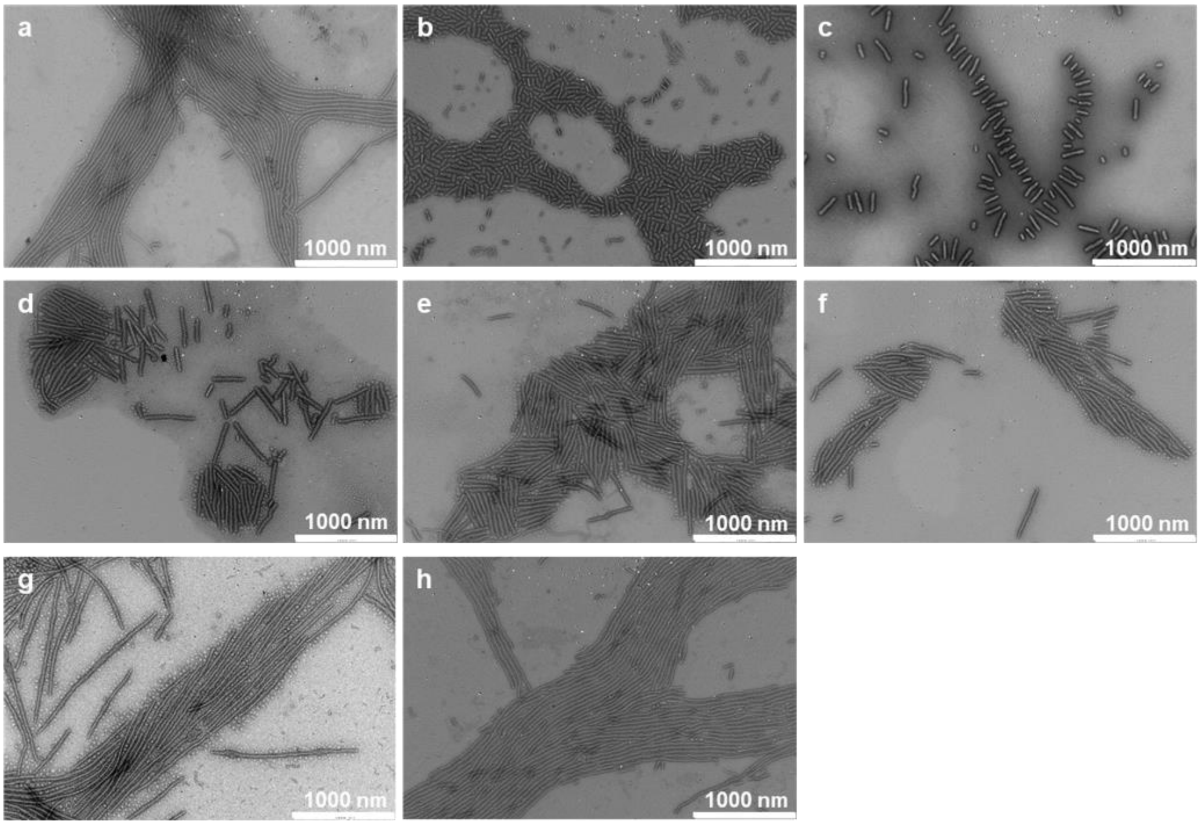

Figure S8. TEM micrographs of (**a**) polydisperse cylinders, (**b**) seeds, (**c**) 250 nm cylinders, (**d**) 500 nm cylinders, (**e**) 750 nm cylinders, (**f**) 1000 nm cylinders, (**g**) 1500 nm cylinders, and (**h**) 2000 nm cylinders in water. 1% uranyl acetate was used as a negative stain. Scale bar = 1 μm.

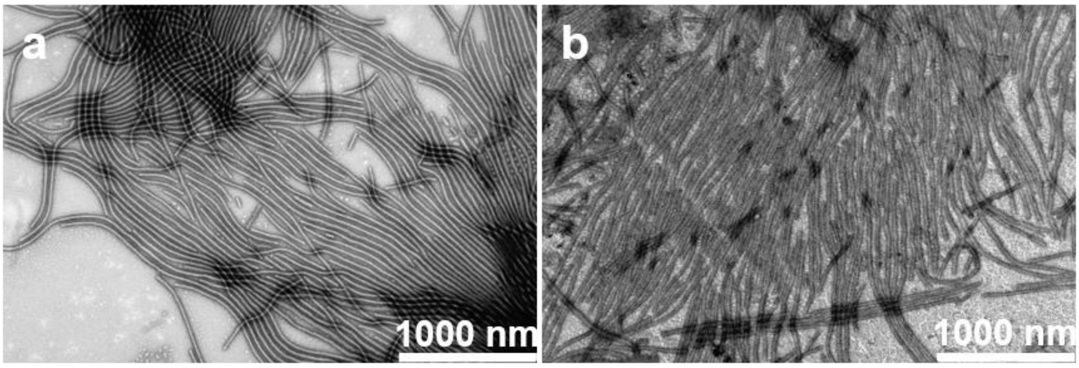

Figure S9. TEM micrographs of (**a**) cylinders before stirring and vortexing and (**b**) after stirring and vortexing. 1% uranyl acetate was used as a negative stain. Scale bar = 1 μm.

Mechanical properties characterization

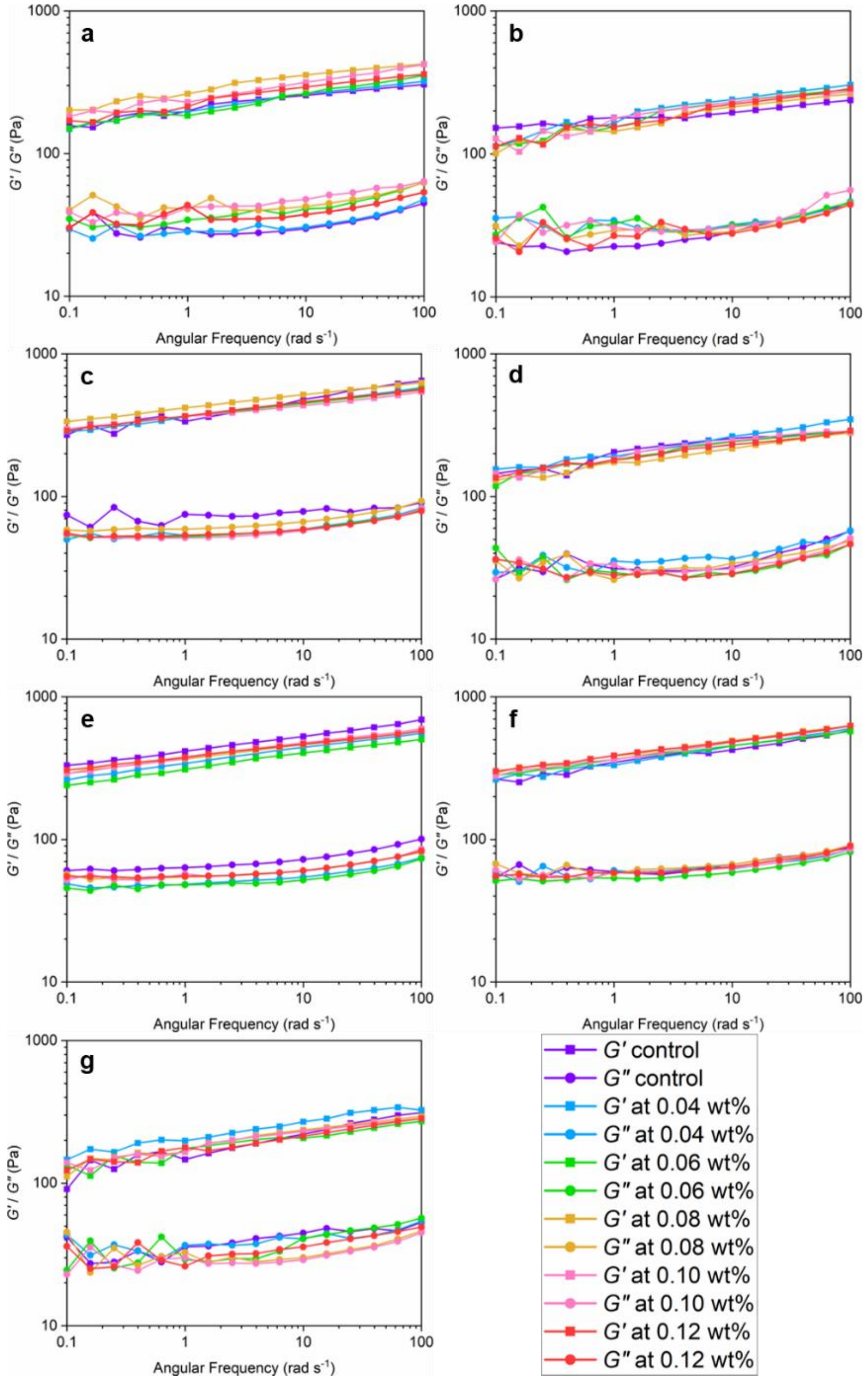

Figure S10. Dynamic oscillatory frequency sweeps at 0.5% strain of calcium-crosslinked alginate hydrogels at 0.50 eq. calcium with 0 wt% (control), 0.04 wt%, 0.06 wt%, 0.08 wt%, 0.10 wt%, and 0.12 wt% PCL_{50}-*b*-$PMMA_{20}$-*b*-$PDMA_{200}$ cylindrical micelles with different lengths, which included (**a**) 65 nm, (**b**) 250 nm, (**c**) 500nm, (**d**) 750 nm, (**e**) 1000 nm, (**f**) 1500 nm, and (**g**) polydisperse cylinders.

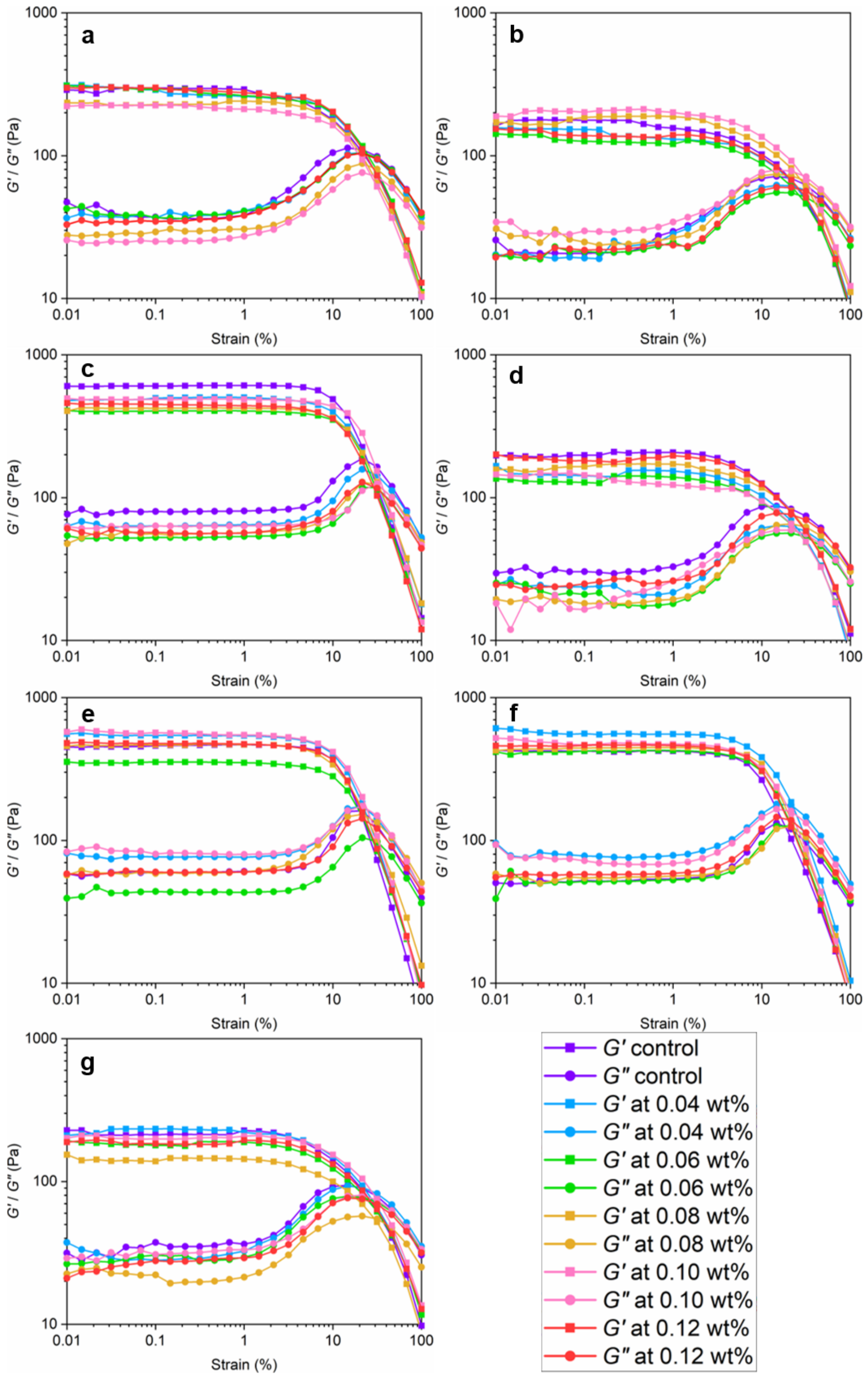

Figure S11. Strain-dependent oscillatory rheology measurements at 10 rad s^{-1} angular frequency (ω) of calcium-crosslinked alginate hydrogels at 0.50 eq. calcium with 0 wt% (control), 0.04 wt%, 0.06 wt%, 0.08 wt%, 0.10 wt%, and 0.12 wt% PCL_{50}-*b*-$PMMA_{20}$-*b*-$PDMA_{200}$ cylindrical micelles with different lengths, which included (**a**) 65 nm, (**b**) 250 nm, (**c**) 500nm, (**d**) 750 nm, (**e**) 1000 nm, (**f**) 1500 nm, and (**g**) polydisperse cylinders.

Table S2 Strain values at the flow point for nanocomposite alginate hydrogels enriched with different concentrations of PCL_{50}-*b*-$PMMA_{20}$-*b*-$PDMA_{200}$ cylinders with different lengths. Data are presented as average ± standard deviation.

**Nanoparticle content (wt%)**	**238 nm**	**500 nm**	**748 nm**	**1428 nm**	**2108 nm**	**Polydisperse**	**Spheres**
**0**				20.9 ± 1.9			
**0.04**	23.9 ± 1.8	24.2 ± 0.5	25.1 ± 1.5	25.3 ± 0.8	23.0 ± 2.6	23.0 ± 1.0	23.0 ± 0.6
**0.06**	24.6 ± 1.0	26.6 ± 1.2	30.0 ± 2.0	27.4 ±1.1	27.2 ± 1.6	22.4 ± 1.1	24.6 ± 0.3
**0.08**	25.5 ± 1.0	27.0 ± 1.2	33.8 ± 1.8	30.3 ± 0.5	23.2 ± 1.3	22.4 ± 1.4	30.4 ± 2.8
**0.10**	27.8 ± 1.7	29.0 ± 2.1	36.8 ± 2.3	26.1 ± 1.3	24.4 ± 1.2	21.9 ± 1.4	32.9 ± 3.7
**0.12**	26.7 ± 1.6	26.6 ± 0.1	28.7 ± 3.4	23.3 ± 0.5	23.9 ± 2.0	22.1 ± 1.2	27.4 ± 2.5

Three gels per sample were measured, each gel was tested a minimum of three times.
